# Functional and Molecular Characterization of the *Halomicrobium* sp. IBSBa Inulosucrase

**DOI:** 10.3390/microorganisms9040749

**Published:** 2021-04-02

**Authors:** Gülbahar Abaramak, Jaime Ricardo Porras-Domínguez, Henry Christopher Janse van Rensburg, Eveline Lescrinier, Ebru Toksoy Öner, Onur Kırtel, Wim Van den Ende

**Affiliations:** 1 IBSB-Industrial Biotechnology and Systems Biology Research Group, Bioengineering Department, Marmara University, Istanbul 34722, Turkey; gulbahar.abaramak@gmail.com (G.A.); ebru.toksoy@marmara.edu.tr (E.T.Ö.); 2 Laboratory of Molecular Plant Biology, KU Leuven, Kasteelpark Arenberg 31, 3001 Leuven, Belgium; jaimerporras@hotmail.com (J.R.P.-D.); henry.jansevanrensburg@unibas.ch (H.C.J.v.R.); 3 Laboratory for Medicinal Chemistry, Rega Institute for Medical Research, Herestraat 49, P.O. Box 1041, 3000 Leuven, Belgium; eveline.lescrinier@kuleuven.be

**Keywords:** fructan, inulin, inulosucrase, Haloarchaea

## Abstract

Fructans are fructose-based (poly)saccharides with inulin and levan being the best-known ones. Thanks to their health-related benefits, inulin-type fructans have been under the focus of scientific and industrial communities, though mostly represented by plant-based inulins, and rarely by microbial ones. Recently, it was discovered that some extremely halophilic Archaea are also able to synthesize fructans. Here, we describe the first in-depth functional and molecular characterization of an Archaeal inulosucrase from *Halomicrobium* sp. IBSBa (*Hmc*Isc). The *Hmc*Isc enzyme was recombinantly expressed and purified in *Escherichia coli* and shown to synthesize inulin as proven by nuclear magnetic resonance (NMR) analysis. In accordance with the halophilic lifestyle of its native host, the enzyme showed maximum activity at very high NaCl concentrations (3.5 M), with specific adaptations for that purpose. Phylogenetic analyses suggested that Archaeal inulosucrases have been acquired from halophilic bacilli through horizontal gene transfer, with a HX(H/F)T motif evolving further into a HXHT motif, together with a unique D residue creating the onset of a specific alternative acceptor binding groove. This work uncovers a novel area in fructan research, highlighting unexplored aspects of life in hypersaline habitats, and raising questions about the general physiological relevance of inulosucrases and their products in nature.

## 1. Introduction

For a very long time, the Archaea superkingdom in the tree of life remained a mystery, with a focus on isolates from extreme environments (e.g., extreme heat, salt or acidity), like *Pyrolobus fumarii* holding the upper temperature limit for life (113 °C; [1]), *Haloquadratum walsbyi*, an extreme salt loving photosynthetic organism [2] and *Picrophilus* spp. capable of growing around pH 0 [3]. Nonetheless, the fact that the specific requirements of Archaea are difficult to establish in a lab environment hampered research progress on these intriguing organisms for a long time [4]. However, the genomics revolution revealed that Archaea, considered as a separate superkingdom, are more widespread than originally thought. We have learned from metagenomics that their occurrence is not limited to extreme environments, and an impressive diversity of Archaea was lately discovered in an array of moderate environments as well [5]. However, their specific functions in microbial communities of such environments remain puzzling.

Hitherto, there had not been much hard evidence that the Archaea superkingdom harbours genuine pathogenic organisms according to Koch’s disease postulates [6]. On the contrary, their surprisingly abundant presence in the human gut system [7] and in the endophytic context of stressed plants [8,9] points at a possible role in an overall balancing of microbial communities in such a way that beneficial, rather than detrimental, effects are generated for the host. The Archaean dominance is even more clear in extreme environments such as salt lakes [10] and acid lakes [11], where the microbial diversity is limited through the extreme environments and the low overall abundancy of nutrients. In many of these cases, carbon inputs for these Archaea [12] are provided only by a few photosynthetically active organisms (cyanobacteria, algae) [13,14]. One can imagine that symbiotic interactions or associations become even more critical to establish stable microbial communities in such environments like, for instance, the hypersaline Tuz Lake in inland Turkey [15,16]. It was reported that halophilic Archaea of the Haloarchaea class (also known as Halobacteria) operating in such environments recruited genes from halophilic bacteria and, more specifically, genes that enable them to utilize a wide range of carbohydrates via aerobic respiration; something their methanogenic ancestors were unable to do [17].

Microbes can build different types of extracellular carbohydrate polymers in their outer layers as part of their ExoPolymeric Substances (EPS), for purposes of overall protection and/or as a temporal energy reserve [18], although it should be noted that EPS can also be used for interaction with surfaces and/or in building and maintaining biofilms [19], the aggregation of plant root-adhering soil [20] and the adherence of biocontrol organism biofilms to plant roots to counteract disease [21]. A spontaneous mutation revealed the influence of *Lactobacillus johnsonii* EPS on bacteria-host interaction in the context of the gastrointestinal tract [22].

One of the most intriguing classes of carbohydrate-type EPS are the fructans, which are fructose (Fru) polymers built from sucrose (Suc) ( [23] and references therein. We recently published that the “fructan syndrome”, the capacity to synthesize fructans, seems to be restricted to the Haloarchaea [24,25], a subclass adapted to function under extremely high salt stress (3–5 M NaCl). Overall, the origin and distribution of fructan synthesis in the tree of life remains poorly understood, with occurrence in (subsections of) eubacterial, archaean, fungal and plant kingdoms, but not in animals, although fructan breakdown enzymes (glycoside hydrolase 32 members: GH32) may have been transferred to a few insects through horizontal gene transfer (HGT; [26,27]). GH32 levanases have also been reported in the genome of bacteriophages [28].

In fructanogenic microbes, i.e., those that are able to accumulate fructans, two main types of fructans occur: levans (β2,6 linkages; widespread) and inulins (β2,1 linkages; rare) that are produced by single enzymes termed levansucrases and inulosucrases, respectively [29,30]. Typically, the degree of polymerization (DP) of microbial fructans can be 10^3^–10^4^ times longer than those observed in plants, which are also able to produce structurally more diverse neokestose-based fructans [31]. However, microbial sucrases can also produce low DP fructo-oligosaccharides (FOS) of the inulin- or levan-type in various proportional ratios with their high DP fructans, depending on the enzymes and specific conditions [32]. Levansucrases and inulosucrases belong to the family 68 of glycoside hydrolases (GH68), while plant fructosyl transferases belong to GH32, showing an extra structural element (sandwich) besides the typical five-bladed β propeller harbouring the catalytic triad [33]. While many 3D structures of levansucrases are available, so far only one structure is available for inulosucrase [34]. All these enzymes and/or fructans produced from their respective source organisms are particularly interesting in terms of industrial applications, including their use as prebiotics, immunomodulators, priming compounds as well as for an array of food and nonfood applications [35,36,37,38,39]. Fructan-producing lactic acid bacteria gained great attention in the fermented food sector, contributing to the increased production of healthy foods [40].

Following the characterization of the first halophilic levansucrase from *Halomonas smyrnensis* (*Hs*Lsc; [25]), and as part of our main research focus on fructanogenic halophiles, where we are trying to elucidate the interplay between fructan production and salinity [41], we previously identified an Archaean strain from Tuz Lake able to produce inulin-type fructans, termed *Halomicrobium* sp. IBSBa (*Hmc*. sp. IBSBa; [16]). We fully sequenced its genome, and report here on the heterologous expression of its single GH68 gene in *Escherichia coli* and the functional and molecular characterization of the heterologous enzyme, an inulosucrase specifically adapted to function under high salt conditions. Following general convention, we refer to this GH68 member as *HmcIsc* (gene) or *Hmc*Isc (protein) from this point onwards.

## 2. Materials and Methods

### 2.1. Microorganisms and Cultivation Conditions

*Hmc*. sp. IBSBa was originally isolated from crude salt samples of Tuz Lake, Turkey [16]. DSMZ Medium 372 (DSMZ-German Collection of Microorganisms and Cell Cultures, Leibniz, Germany) with the following composition was used to cultivate the strain: 200 g/L NaCl, 20 g/L MgSO_4_.7H_2_O, 2 g/L KCl, 3 g/L Na_3_ citrate, 5 g/L casamino acid, 5 g/L yeast extract, 1 g/L glutamic acid, 36 mg/L FeCl_2_.4H_2_O and 0.36 mg/L MnCl_2_.4H_2_O. pH was adjusted to 7.0 and the medium was autoclaved at 121 °C for 15 min. Fifty mL of the medium in a 250 mL Erlenmeyer flask was inoculated with a frozen culture stock at 1% (*v*/*v*) ratio and left to grow at 45 °C and 180 rpm for 4–5 days.

For *Escherichia coli* cultures, Luria-Bertani (LB) medium was prepared with 10 g/L NaCl, 10 g/L peptone and 5 g/L yeast extract. Medium was autoclaved at 121 °C for 15 min. Antibiotics (kanamycin or chloramphenicol) were added after sterilization for the selection of transformants. Cultures were incubated at 37 °C and 180 rpm.

### 2.2. Cloning of HmcIsc

The genomic DNA of *Hmc*. sp IBSBa was extracted using DNeasy^®^ UltraClean^®^ Microbial Kit (QIAGEN, Antwerp, Belgium) following the manufacturer’s instructions and used as a template for PCR amplification of the inulosucrase *HmcIsc* gene. The Whole Genome Shotgun project was deposited at DDBJ/ENA/GenBank under the accession JADPQC000000000. The version described in this paper is version JADPQC010000000. Primers were designed for directional cloning (Table S1). Both C- and N-terminal 6xHis-Tag versions of the same gene were designed and cloned. New England BioLabs Q5 enzyme (NEB, Beverly Hills, MA, USA) with Q5 High-Fidelity 2X Master Mix Kit (NEB, Beverly Hills, MA, USA) was used in PCR. The GH68 family gene sequence for C-terminal and N-terminal 6xHis-Tag were amplified by PCR on 0.2 µg of genomic DNA of *Hmc*. sp. IBSBa in a reaction mixture of 50 µL. The reaction mixture contained 25 µL of 2X Master Mix, 2.5 µL of each forward and reverse primers (10 µM) and 17 µL nuclease-free water. The thermocycling conditions were 30 s initial denaturation at 98 °C in a VWR^®^ PCR Thermal Cycler XT96 (VWR International, Leuven, Belgium), followed by 30 cycles with 10 s denaturation at 98 °C, 30 s annealing at 69 °C for C-terminal/66 °C for N-terminal, and 35 s extension at 72 °C, with a final extension of 2 min at 72 °C. PCR products at expected size and quantity were purified by the Wizard^®^ SV Gel and PCR Clean-up System (Promega, Madison, WI, USA) and then digested with respective restriction enzymes. After digestion, samples were incubated at 80 °C for 20 min to inactivate the enzymes then put on ice, run on a 0.8% agarose gel, and digested bands were cut and subjected to clean-up.

Vector DNA pET-28a(+) and insert DNA (GH68 C-terminal/N-terminal 6xHis-Tag) were ligated in a 20 µL reaction mixture that contained T4 DNA Ligase Buffer (1X), 50 ng vector DNA, 36.79 ng insert DNA and 0.02 U/µL New England BioLabs T4 DNA ligase at final concentrations. Ligation samples were mixed gently and incubated at room temperature for 30 min. Samples were incubated at 16 °C overnight. Heat inactivation was carried out at 65 °C for 10 min, and samples were stored at 4 °C until transformation.

To multiply and store the vector, 5 µL of pET-28a(+) plasmid containing *Hmc*Isc was used to transform Invitrogen One Shot *E. coli* TOP10 Chemically Competent Cells (Invitrogen, Carlsbad, CA, USA). Mixtures were incubated on ice for 30 min, followed by a heat shock at 42 °C for 45 s, and placed back on ice for 2 min. LB medium (250 µL) was added to the cell mixture and incubated at 37 °C (180 rpm; 1.5 h). 100 µL of each transformation was plated onto LB agar plates with kanamycin (50 ng/µL). Plates were incubated overnight at 37 °C. Plasmids were isolated from transformed *E. coli* TOP10 cells using QIAprep Spin Miniprep Kit (QIAGEN, Antwerp, Belgium) following manufacturer’s instructions.

The GH68 family gene sequence with C-terminal and N-terminal 6xHis-Tag sequences were amplified by PCR on 0.17 µg of DNA of isolated pET-GH68C and pET-GH68N in a reaction mixture of 50 µL also containing 1X GoTaq^®^ Buffer (Promega), 0.3 µM GH68 forward primer, 0.3 µM GH68 reverse primer, 0.3 mM dNTPs and 0.02 U/µL Promega GoTaq^®^ DNA Polymerase. The thermocycling conditions were 3 min initial denaturation at 95 °C in a VWR^®^ PCR Thermal Cycler XT96, followed by 30 cycles with 50 s denaturation at 95 °C, 50 s annealing at 63 °C, and 1 min 30 s extension at 72 °C, with a final extension of 10 min at 72 °C. PCR samples were run on 0.8% agarose gel. Successful results were sent to sequencing. Three µL of isolated pET-GH68C and pET-GH68N were used to transform to *E. coli* Rosetta(DE3) competent cells and transformants were selected on LB plates with kanamycin (50 ng/µL) and chloramphenicol (34 ng/µL).

### 2.3. Expression and Purification of HmcIsc

For the production of the *Hmc*Isc inulosucrase, a preculture was prepared by picking a pET-GH68C transformant colony from LB selection agar and transferring it to 5 mL LB medium with kanamycin (50 ng/µL) and chloramphenicol (34 ng/µL), which was incubated at 37 °C and 180 rpm overnight. Two milliliters from the preculture were then transferred to two 200 mL of LB media with kanamycin (50 ng/µL) in 1 L Erlenmeyer flasks. Flasks were incubated at 37 °C and 180 rpm. Gene expression was induced by adding 1 mM of isopropyl β-D-l-thiogalactopyranoside (IPTG) when OD_600_ reached ~0.6. Cells were incubated at 20 °C with 180 rpm shaking overnight. An uninduced culture was used as negative control.

For the purification, cells were centrifuged at 10,000× *g* and 4 °C for 20 min. Pellets were suspended in 5–6 mL of 20 mM potassium phosphate buffer with 300 mM NaCl (pH 6.6). Suspended cells were disrupted by ultrasonication (Bandelin Sonopuls UW 2200) with 28–31% of nominal power, 30 s on, 30 s off, 30 cycles on ice. Cell debris was removed by centrifugation at 8000× *g* for 10 min. Supernatant containing the *Hmc*Isc inulosucrase enzyme was subjected to purification via immobilized metal affinity chromatography (IMAC) using nickel-nitrilotriacetic acid (Ni-NTA) agarose resin according to manufacturer’s instructions (QIAGEN). Briefly, equilibration, wash and elution buffers for IMAC contained 10, 20 and 300 mM imidazole, respectively, in 20 mM potassium phosphate buffer with 300 mM NaCl (pH 7.4). Fractions of 1 mL were collected and checked for protein content and *Hmc*Isc enzyme activity.

### 2.4. SDS-PAGE Analysis

To check the purity and molecular weight of protein fractions, a modified version of the standard sodium dodecyl sulfate-polyacrylamide gel electrophoresis (SDS-PAGE) protocol [42] was followed in a Mini-PROTEAN^®^ Tetra Cell (BIO-RAD, Hercules, CA, USA). Acrylamide concentrations of the separating and stacking gels were 10% and 5%, respectively. Coomassie Brilliant Blue R-250 was used to visualize the protein bands. PeqGOLD ProteinMarker I (VWR International, Leuven, Belgium) was used as the protein ladder.

### 2.5. Protein and Enzyme Activity Assays

Protein concentrations of the collected fractions were determined by measuring their absorbance at 280 nm via a BIO-RAD SmartSpec Plus spectrophotometer. According to ExPASy ProtParam, an A_280_ value of 1 g/L of *Hmc*Isc is 1.7 (molar extinction coefficient: 83,435 M^−1^ cm^−1^). Protein concentrations were calculated by using this molar extinction coefficient. To determine *Hmc*Isc enzyme activity, one volume of enzyme fraction was incubated in nine volumes of 50 mM potassium phosphate buffer (pH 7.0) with 0.15 M Suc and 3.5 M NaCl at 45 °C for 24 h. Activity of the enzyme was determined by measuring the concentration of released reducing sugars by the 3,5-dinitrosalicylic acid (DNS) method [43]. One unit of the enzyme activity (U) was defined as the amount of enzyme required to release 1 µmol of reducing sugars per minute. All enzyme activity reactions in this study were run in triplicates.

### 2.6. Effects of Salt Type and Salt Concentration

Enzymatic reactions were carried out as described above with NaCl, KCl, KI, NaBr, MgCl_2_ or MgSO_4_ to assess the effects of different salts at 3.5 M concentration each. To optimize salt concentration, enzyme activities of reactions with seven different NaCl concentrations between 0 M and 4.5 M were determined in the presence of 50 mM potassium phosphate buffer with 0.15 M Suc (pH 7.0). All reactions were incubated at 45 °C. Enzyme activities were determined via the DNS method at 24 h.

### 2.7. Effects of pH and Temperature

Enzymatic reactions were carried out at nine different pH values ranging from pH 4.0 to pH 9.0 in the presence of 0.15 M Suc and 3.5 M NaCl. 50 mM sodium acetate buffer for pH 4 and pH 5, 50 mM potassium phosphate buffer for pH 6 to pH 7.5, 50 mM Tris buffer for pH 8.0 and pH 8.5 and 50 mM carbonate-bicarbonate buffer for pH 9.0 were used. Reactions were carried out at 45 °C for 24 h. To analyze the effect of temperature, standard reactions were incubated at different temperatures (4, 15, 37, 45, 50, 55, 60, 65, and 70 °C). Enzyme activities were determined with DNS method at 24 h.

### 2.8. Effects of Various Substances

Effects of various divalent cations (Ba^2+^, Fe^2+^, Ca^2+^, Cu^2+^, Co^2+^, Ni^2+^, Mg^2+^, Mn^2+^), detergents (SDS and Triton X-100) and EDTA on enzyme activity at 1 mM and 5 mM final concentrations each were investigated. Divalent cations were added to reactions as BaCl_2_, FeSO_4_, CaCl_2_, CuSO_4_, CoSO_4_, NiCl_2_, MgSO_4_, MnCl_2_, respectively, to give final metal ion concentrations of 1 mM and 5 mM. Standard enzymatic reactions were run to assess activity.

### 2.9. Enzyme Activity over Time

A standard enzymatic reaction was carried out for 600 h with samples taken regularly. Released total reducing sugar concentrations were determined with the DNS method.

### 2.10. Enzyme Kinetics

Kinetics were determined by considering a 0–500 mM Suc concentration range. Reactions were carried out in 50 mM potassium phosphate buffer with 3.5 M NaCl and varying Suc concentrations (pH 7.0) at 45 °C for 24 h. The DNS method was used to determine enzyme activities. GraphPad Prism5 software (GraphPad Prism version 7.0.0 for Windows, GraphPad Software, San Diego, CA, USA) was used to obtain a kinetic model.

### 2.11. Fructan Production and Purification

A standard enzymatic reaction in a total volume of 8 mL was carried out in 50 mM potassium phosphate buffer with 3.5 M NaCl and 0.15 M Suc (pH 7.0) at 45 °C for 4–5 days. The mixture was boiled at 90 °C for 5 min to denature the *Hmc*Isc enzyme and cooled on ice. Two volumes of ice-cold ethanol were added to the mixture and mixed immediately for precipitation overnight at −20 °C, followed by centrifugation at 8000× *g* for 10 min. The pellet still contained some small sugars and precipitated salts, which were progressively removed by five freeze/thaw cycles in 20% ethanol. The pellet was dissolved in 100 µL water and ethanol was mostly removed through Speedvac.

### 2.12. NMR Characterization of the Fructan

Nuclear Magnetic Resonance (NMR) spectroscopy was applied as described [16] to determine the nature of the produced fructans, either inulin or levan-type. The 1D proton spectrum was recorded at Bruker Avance II 500 MHz at 293 K equipped with 5 mm TXI Z gradient probe. In one-dimensional proton spectra, the HOD signal was suppressed by applying presaturation during a relaxation delay for 1 s.

### 2.13. Molecular and Structural Analyses

The homology model of the *Hmc*Isc inulosucrase was created through the multitemplate protein structure prediction tool I-TASSER [44,45,46] using the templates of the crystal structures with the following Protein Data Bank (PDB) codes: 6FRW, 4D47, 3VSR and 1W18. The model with the highest score was selected for further steps. The 365–380 amino acid loop, not present in any of the templates, was modeled using the Molecular Operating Environment (MOE) loop prediction tool, generating thousand conformations and the best conformation with the lowest overall energy level was selected. The complete homology model was further minimized with a layer of explicit solvent covering 10 Å, adding 4 M NaCl, and protonated at pH 7.0. The homology model was minimized using Amber10/14EHT as the force field, with a gradient of 0.1 Kcal/mol/A2 (MOE, 2019). The latter software was also used for an in-silico mutagenesis of the cysteine bridges at 4 M NaCl and pH 7.0 through a dynamic method for 50 ps. The sugar molecules used were constructed using a Glycam server (www.glycam.org, accessed date: 1 February 2021; [47]) and modified in the case of the glycosylation chain using MOE. The molecular docking analysis was performed using GOLD-Protein Ligand docking software [48] using Chemscore as a score function. The binding site was defined in the donor mode’s nucleophile position and the fructosyl intermediate in the acceptor mode, selecting all the amino acids flexible in a radius of 15 Å The fructosyl intermediate was generated by binding an α-ᴅ-fructofuranose molecule to the nucleophile using GOLD covalent docking. The fructosyl intermediate model was minimized with the same procedure as explained before. One hundred solutions were generated per analysis and the complexes with the highest scores and lowest energies were selected. The interactions were detected by MOE.

The model with the glycosylation chain was created through GOLD covalent docking, minimized and analyzed with MOE. The top five solutions were analyzed to find all the amino acids interacting with the glycosyl residues. All figures were prepared using PYMOL 2.4.

### 2.14. Phylogenetic Analysis

Nucleotide sequences from fourteen species of systematically important Haloarchaea, Gram-positive and Gram-negative bacteria were selected from NCBI for the family GH68 and the well-known molecular marker 16S rRNA. The sequences were downloaded manually, converted to FASTA files and aligned in Clustal W [49]. The alignments were deduced in BioEdit v.7.0.0 [50]. Neighbor Joining trees were produced in ClustalW using the full tree function. The topology in the resulting GH68 molecular phylogenetic tree indicates HGT. To further examine this reticulate event, phylogenetic networks were generated in SplitsTree4 v4.13.1 [51]. For ideal data, this method gives rise to a tree, whereas less ideal data are represented by a tree-like network indicating different and conflicting phylogenies. A matrix was calculated based on the JukesCantor model of DNA evolution, and further used to calculate the distances between pairwise sequences. The resulting matrix was plotted as a NeighborNet splits network to visualize evolutionary relationships and allow for comparison with the molecular phylogenetic trees. The distances in the networks reflect the mean branch length, and the boxes in the middle suggest conflict in the data, in this case HGT. The splits were transformed using reticulate equal angle, and distinct groups were identified based on a balance between maximizing edge weighting, whilst minimizing contradictory splits.

## 3. Results and Discussion

### 3.1. ORF Analysis of the Halomicrobium sp. IBSBa GH68 Member

After isolation of different fructan producing *Halomicrobium* strains [16], the genome of the inulin producing IBSBa strain was fully sequenced. Protein encoding open reading frames (ORFs) were derived, and only a single GH68 member, a predicted inulosucrase, was detected. The observation that only a single GH68 member is detected per genome is rather common among the other fructanogenic Haloarchaean organisms that have been identified so far (www.cazy.org/GH68_archaea.htmL, Access Date: 01/02/2021), although there are exceptions. It should be noted that not all Haloarchaean species are fructanogenic though, suggesting that other types of EPS may be able to take over the function of fructans [16,23]. However, it seems that glucans are not among those, since glucansucrases (GH70; [52]) could not yet be identified in Haloarchaea (www.cazy.org/GH70.htmL, Access Date: 01/02/2021). This indicates that fructans, rather than glucans, play important roles in salt-loving organisms, as suggested before [25].

The predicted ORF of *HmcIsc* (1305 bp including the stop codon) encodes the *Hmc*Isc inulosucrase enzyme consisting of 434 amino acids with a predicted MW of 48.2 kDa and a isoelectric point (pI) of 4.58. Two putative N-glycosylation sites (NGS and NDS) were identified (see multiple alignment below). *Hmc*Isc is a highly negatively charged/hydrophilic enzyme, in line with other extracellular haloenzymes [41]. The number of negatively charged residues is 70 (D: 43; E: 27), while the number of positively charged residues is 37 (K: 2; R: 35). Interestingly, the ratio of anionic to cationic residues for *Hmc*Isc (1.94) is similar to the one observed for the halophilic levansucrase for the bacterium *H. smyrnensis* (2.13) but much higher than the one recorded for *Bacillus subtilis* levansucrase (1.10). Strikingly, the K/R ratio differed strongly between *Hmc*Isc (0.06) and the bacterial levansucrases from *H. smyrnensis* (1.14) and *B. subtilis* (4.78), respectively. Although no signal peptide could be detected with SignalP 5.0 [53], the PRED-TAT server [54] predicted a clear signal peptide (reliability score: 0.875), as it also did for SlaA that served as positive control (reliability score 0.925). SlaA is the best characterized extracellular S-layer protein from the thermoacidophilic archaeon *Sulfolobus acidocaldarius* [55]. This predicted extracellular localization of *Hmc*Isc is in full accordance with the previously reported purification of inulin-type fructans from the Suc supplemented growth medium of *Hmc*. sp. IBSBa [16].

### 3.2. Phylogenetic Analysis: Evidence in Favour of HGT

*Halomicrobium* spp. classify within the domain of the Archaea, kingdom Euryarchaeota, Stenosarchaea group, class Halobacteria, order Halobacteriales, family Haloarculaceae (https://www.ncbi.nlm.nih.gov/Taxonomy/Browser/wwwtax.cgi, accessed date: 8 January 2021; [56]). Overall, the capacity to produce fructan seems to have arisen multiple times during evolution. The polyphyletic origin of fructan biosynthesis in nature [57] combined with the possible occurrence of HGT [17] leads to complexities to keep the overall overview throughout the tree of life. It is now generally accepted that Archaea evolved from bacteria and not the other way around as originally thought for a very long time. The finding that fructans only occur within the Haloarchaea [16], and not in any other section of the Archaea, is very intriguing. Recent insights on the archaeal phylum Verstraetearchaeota within the Thaumarchaeota, Aigarchaeota, Crenarchaeota and Korarchaeota (TACK) superphylum [58] strengthen the notion that the last universal common ancestor from Archaea and Bacteria was a CO_2_-reducing anaerobic methanogen [59], possibly a thermophilic one. Fructan synthesis, depending on Suc associated with oxygenic autotrophy [60], has never been reported in methane-producing organisms. Moreover, the occurrence of fructan is very rare in thermophilic bacteria [61] and not known to occur in present day nonhalophilic Archaea. It was reported that HGT from halophilic bacteria was a prominent event during adaptation processes to high salt conditions in the Haloarchaea lineage [17]. Here, we propose that this may also be the case for fructan synthesis associated with GH68 inulosucrase activity.

**Figure 1:**
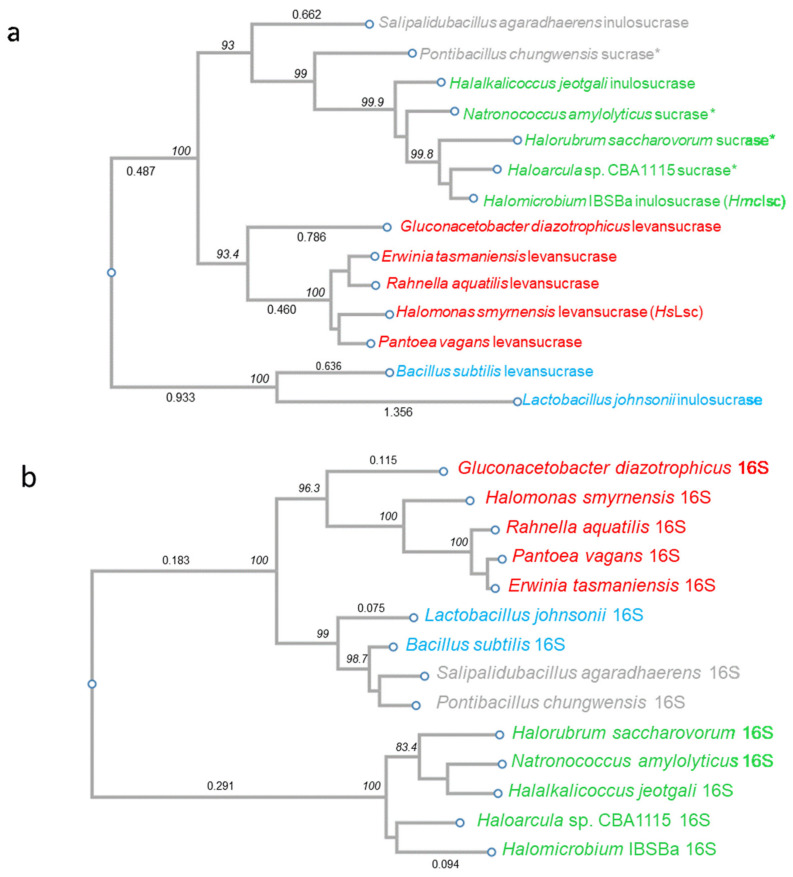
(**a**) Phylogenetic tree based on the protein sequences of GH68 sucrases from selected organisms. These include GH68 sucrases from Gram-positive species, including the inulosucrases from *S. agaradhaerens* (grey; MF422638.1), the putative inulosucrase from *P. chungwhensis* (grey; NZ_AVBG01000018.1), the levansucrase from *B. subtilis* (blue; AY150365.1) and the inulosucrase from *L. johnsonii* (blue; AE017198.1). Gram-negative GH68 sucrases (red) include those of the *G. diazotrophicus* levansucrase (AY853402.1), the *E. tasmaniensis* levansucrase (X75079.1), the *R. aquatilis* levansucrase (AY027657.1), the *H. smyrnensis* levansucrase (KC480580.1) and the predicted levansucrase from *P. vagans* (CP014127.2). Among the Haloarchaea (green), the *H. jeotgali* inulosucrase (CP002062.1), the *N. amylolyticus* putative inulosucrase (NZ_AOIB01000038.1), the predicted inulosucrase from *H. saccharovorum* (NZ_AOJE01000020.1)*,* the putative inulosucrase from *Haloarcula* sp. CBA1115 (CP010529.1) and *Hmc*Isc (this work) are depicted. *indicates that the inulosucrase functionality has not yet been proven. (**b**) Phylogenetic tree based on the DNA sequences of 16S rDNA from selected microorganisms. These are derived from Gram-negative bacteria (red) including those of *G. diazotrophicus* (NR_027591.1), *H. smyrnensis* (NR_115697.1), *R. aquatilis* (MN826572.1), *P. vagans* (CP038853.1) and *E. tasmaniensis* (NR_074869.1). Sequences from Gram-positive bacteria (include those of *L. johnsonii* (blue; LIGY000026.1), *B. subtilis* (blue; MT605348.1), *S. agaradhaerens* (grey; FN432808.1) and *P. chungwhensis* (grey; NR_025812.1). Among the Haloarchaea (green), *Haloarcula* sp. CBA1115 (LC198783.1), *Halomicrobium* IBSBa (MK208532), *H. saccharovorum* (NR_113484.1), *H. jeotgali* (LT634701.1) and *N. amylolyticus* (NR_028217.1) are considered. The two species in grey changed position between the GH68 (**a**) and 16S (**b**) phylogenetic trees. Bootstrap values (italic, %) are indicated. A number of branch length labels are indicated (not italic).

A BLASTn was performed with the *HmcIsc* ORF. Besides a list of expected (putative) Haloarchaean GH68 sucrases such as those from *Haloarcula* sp. CBA1115, *Halorubrum saccharovorum* [29], *Natronococcus amylolyticus* [29] and *Halalkalicoccus jeotgali* [30], as well as two bacterial GH68 members (from *Pontibacillus chungwhensis* and *Salipalidubacillus agaradhaerens*), showed surprisingly high homology. Together with a series of selected structurally or functionally characterized sucrases from Gram-positive and Gram-negative origin, a phylogenetic tree was produced (Figure 1a) and fully compared to a phylogenetic tree for 16S derived from the same species (Figure 1b). While the 16S phylogenetic analysis confirmed the expected clear separation between Gram-positive (blue, Figure 1b), Gram-negative (red, Figure 1b) and Archaea (green, Figure 1b), in the GH68 phylogenetic analysis the Gram-positive sucrases of *P. chungwhensis* and *S. agaradhaerens* joined the Archaean upper group, already indicative for HGT. To generate more evidence, phylogenetic networks were also generated in SplitsTree4 v4.13.1 both for GH68 and 16S (Figure S1a,b; [51]). The distances in the networks reflect the mean branch length, and the boxes in the middle demonstrate a conflict in the data, pointing at HGT in the case of GH68. From this analysis, it can be hypothesized that a last common ancestor of *P. chungwhensis* and *S. agaradhaerens* transferred a GH68 gene to a Haloarchaean progenitor. This must be further analyzed in depth in the future, perhaps also allowing an estimation of the timing at which this HGT occurred. This analysis suggests that the transferred gene could have been an inulosucrase. By extension, it can be hypothesized that the whole upper group in Figure 1a may represent a clan of inulosucrases, despite the fact that one study [29] standardly considered some of them as ‘levansucrases’, but without providing any proof of the generated product from Suc. Thus, our data indicate that inulosucrases are more widely distributed in the tree of life than generally assumed. Likely, the historical focus on levansucrases also led gene annotators standardly predicting a levansucrase nature, while many of those may actually represent inulosucrases. However, the other way around, a GH68 member that was predicted to be an inulosucrase, based on phylogenetic and structural information, turned out to be a levansucrase [62]. It can be concluded that our current structure-function insights are still too limited to properly discriminate between inulosucrases and levansucrases, although recent progress has been made on the kestose regioselectivity [63]. In the next two sections, we confirm that *Hmc*Isc indeed represents an inulosucrase.

### 3.3. Cloning, Heterologous Expression and Purification

PCR amplification was performed from genomic DNA, followed by sticky end cloning into the pET-28a(+) vector, with N- (pET-GH68N) and C-terminal (pET-GH68C) His-tagged versions. Heterologous expression in *E. coli* was carried out with the Rosetta(DE3) strain, as advised [30], for the *Halalkalicoccus jeotgali* B3T inulosucrase.

Sucrase activities are most often followed by quantifying the formation of glucose (Glc). Preliminary expression in *E. coli* of pET-GH68N and pET-GH68C showed that crude extracts of the former showed no enzymatic activity (Glc production from Suc). Therefore, for upscaling and purification, GH68C was selected. Growth and expression in *E. coli* occurred as described [25], comparing IPTG-induced and uninduced (negative control) cells. Induced cell extract was subsequently purified by IMAC (Table S2) and exhibited protein bands of *Hmc*Isc right above the 45 kDa ladder on SDS-PAGE (Figure S2). Although 45% of the total activity was lost, a 51.4-fold purification was obtained with specific activities increasing from 1.1 U/g protein to 56.5 U/g protein in the IPTG-induced condition, while very low total and specific activities were recorded in the uninduced cell extract (Table S2). For comparison, only a 4.9-fold purification was obtained in case of the halophilic levansucrase from *H. smyrnensis* AAD6^T^ (*Hs*Lsc; [25]).

### 3.4. Product Identification through NMR

**Figure 2:**
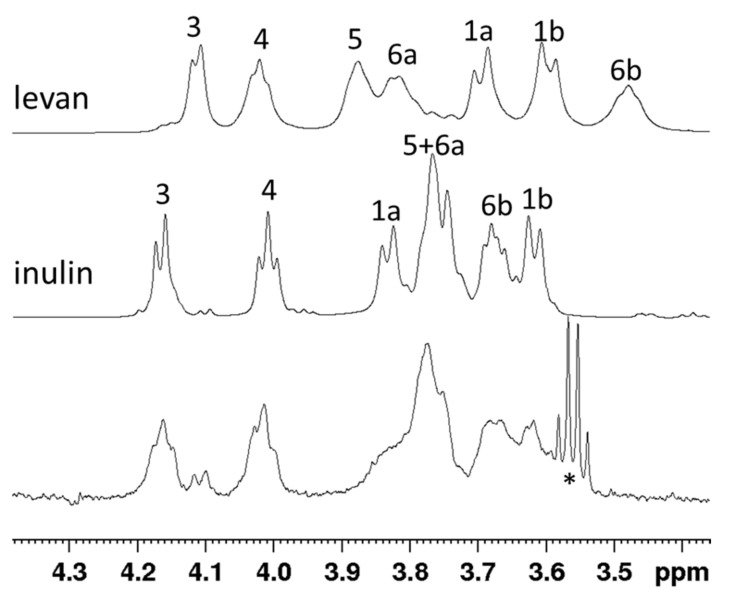
Nuclear magnetic resonance (NMR) results of purified Archaeal fructan produced by *Hmc*Isc. Stacked plot of the assigned 1D ^1^H spectra of two reference samples, levan and inulin, and the produced fructan. The methylene group in the trace of ethanol gives rise to the well-resolved quartet indicated with *.

From preliminary IBSBa growth experiments we learned that neutral to mild alkaline conditions and temperatures between 37 and 45 °C are suitable growth conditions (data not shown). We reasoned that such conditions would also be suitable for the extracellular sucrase under study. Therefore, the purified recombinant *Hmc*Isc was incubated together with 150 mM Suc at pH 7.0 at 45 °C and Glc production was followed. The produced fructan after four days was ethanol precipitated, further purified and dissolved in water prior to NMR analysis. The 1D proton spectra of the fructans produced by the recombinant *Hmc*Isc enzyme were compared to previously-reported spectra on inulin (Orafti^®^ HP, Beneo-Orafti, Oreye, Belgium) and *H. smyrnensis* levan reference samples [16]. Slow tumbling of the high molecular weight fructan strongly broadened the NMR signals, though the chemical shift of signals matched the spectrum of inulin, while the signature signal at 3.5 ppm for levan (proton 6b) was absent in the spectrum of the studied sample (Figure 2). Thus, the *Hmc*Isc produced inulin-type fructans from Suc, as expected, since *Halomicrobium* sp. IBSBa cultures produced inulin [16].

### 3.5. Enzymatic Characterization

While the recently reported Haloarchaean inulosucrase was not a subject of enzymatic characterization [30], here, the purified *Hmc*Isc enzyme was characterized comprehensively in terms of the effects of pH, temperature, salt and heavy metals on the enzyme activity as well as its kinetics by following Glc formation from Suc.

#### 3.5.1. Effects of pH and Temperature

**Figure 3:**
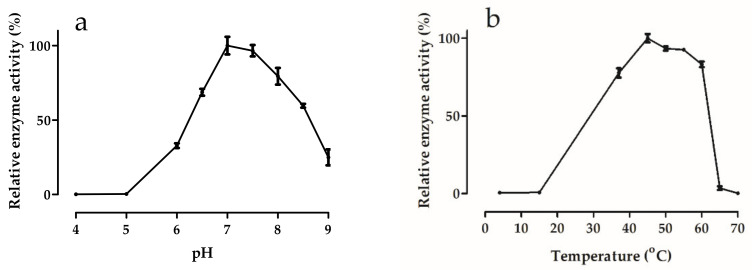
Effects of (**a**) pH and (**b**) temperature on *Hmc*Isc activity. Error bars represent standard error values for three replicates. Activities are expressed as a percentage of the maximal activity. An optimal T of 45 °C was chosen for the pH optimization. An optimal pH of 7.0 was selected for the T optimum determination. Sucrose and salt (NaCl) concentrations were 150 mM and 3.5 M, respectively, and reaction time was 24 h.

In line with the neutral to moderate alkaline pH in Tuz Lake [64], the pH optimum of *Hmc*Isc (7.0–7.5) was situated in this region, with rapid activity loss in the acidic region (Figure 3a). Such a pH optimum is rather unusual among the inulosucrases so far characterized [65], suggesting it may be an adaptation towards the specific environmental conditions in certain salt lakes, such as Tuz Lake. Most characterized inulosucrase enzymes show a more acidic pH optimum, with the exception of that of *S. agaradhaerens* [66] which shows a very broad pH optimum between 6 and 10, and the *Bacillus sp.* 217C-11 inulosucrase, with a pH optimum between 7 and 8 [67]. Similarly, the pH optimum of most levansucrases is also mostly slightly acidic [41] and references therein], also explaining why the discovery of new levansucrases is standardly performed at pH 6.0 [29]. Interestingly, a remarkable shift towards increased activities in the alkaline pH regions was achieved through the generation of cross-linked enzyme aggregates of *Limosilactobacillus reuteri* 121 inulosucrase [68]. This enzyme, with a temperature (T) optimum around 50 °C, is very interesting for use in an industrial context. *Hmc*Isc showed a T optimum which is rather high (45 °C) and broad (between 40 °C and 60 °C) (Figure 3b). In general, a wide variation in T optima can occur among GH68 sucrase enzymes, although many are situated around 30 °C [69]. It should be noted that increased temperatures most often enhance the hydrolase/transferase ratio of GH68 sucrases [70], except in truly thermophilic microorganisms [41]. Hence, the T optimum for maximal fructan production is mostly lower, and methodologies that only detect reducing sugars (hexoses, Fn only FOS) are not adequate to predict temperature-dependent fructan production maxima [71].

#### 3.5.2. Effects of Salt Type and Concentration

**Figure 4:**
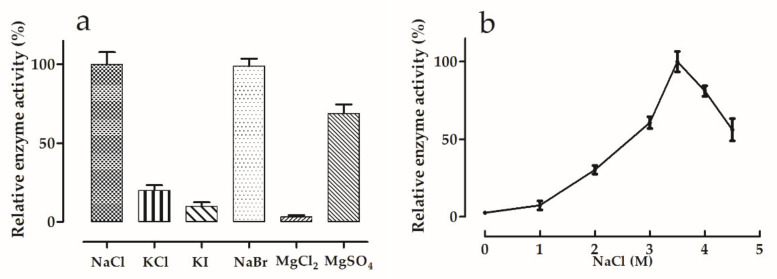
Effects of (**a**) different salt types at 3.5 M concentrations and (**b**) varying NaCl concentrations on *Hmc*Isc activity. Error bars represent standard error values for three replicates. Activities are expressed as a percentage of the maximal activity. An optimal T of 45 °C and an optimal pH of 7.0 were chosen for the salt optimization. Sucrose concentration was 150 mM, and reaction time was 24 h.

The main ions in Tuz Lake are Na^+^ (114.25 g/L) and Cl^−^ (201.67 g/L), being much higher than SO_4_^2−^ (19.85 g/L), Mg^2+^ (11.44 g/L), K^+^ (6.55 g/L) and Ca^2+^ (0.32 g/L) [64]. When comparing the effect of the salt type on the *Hmc*Isc activity (Figure 4a), it is striking that the K^+^ salts resulted in a tremendous inhibition as compared to NaCl, suggesting that the Na^+^ ion, being the most abundant in Tuz Lake, is one of the most essential factors to preserve the enzyme stability and function. In line with this view, NaBr and NaCl showed similar enzyme activities. Accordingly, replacement of Na^+^ by Mg^2+^ in the chlorine salt led to a drastic decrease in *Hmc*Isc activity. However, replacing Cl^−^ by SO_4_^2−^ restored this inhibition to a great extent (Figure 4a). Curiously, *Hs*Lsc depended much less on Na^+^ since 86% of its activity was retained when replacing NaCl by KCl. An even more striking difference between *Hs*Lsc and *Hmc*Isc is that the latter was not affected by NaBr, while the former was 100% inhibited [25]. *Hmc*Isc activity increases with increasing NaCl concentration (Figure 4b), reaching an optimum at 3.5 M NaCl. At 2 M NaCl, generating toxic effects on many mesophilic enzymes, this halophilic enzyme only retained about 30% of its maximum activity. Depending on the season and rainfall (the lake completely dries in summer), Tuz Lake NaCl concentrations may vary between 3 M and full saturation (approx. 5.5 M), fitting well with the optimal salt concentrations required for *Hmc*Isc functioning in vitro (Figure 4b). It should be noted that *Hmc*Isc is not irreversibly denatured by a temporal exposure to lower salt, since purification on the IMAC occurred at 0.3 M NaCl.

#### 3.5.3. Effects of Various Substances

Table 1 describes the effects of SDS, Triton X-100, EDTA and an array of metal ions at 1 mM and 5 mM. SDS and Triton X-100 appeared strong inhibitors, in contrast to EDTA, which slightly induced the activity, perhaps through sequestering divalent metals. Accordingly, the divalent metals Cu^2+^, Co^2+^ and Ni^2+^ showed detrimental effects, and Ca^2+^, though an important cofactor for several GH sucrases [72], inhibited *Hmc*Isc. Fe^2+^ progressively inhibited activity at enhanced concentrations. Remarkably, *Hmc*Isc was not inhibited by Ba^2+^, in contrast to *Hs*Lsc which was completely inhibited by 5 mM Ba^2+^.

#### 3.5.4. Enzyme Kinetics

**Figure 5:**
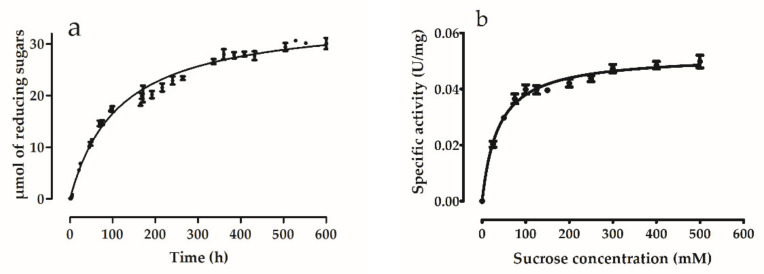
Activity of *Hmc*Isc assessed (**a**) temporally by following µmol of released reducing sugars, and (**b**) at increasing sucrose concentrations. Error bars represent standard error values for three replicates. µmol concentrations of released reducing sugars were followed with 150 mM initial sucrose concentration. Activity of *Hmc*Isc assessed at increasing sucrose concentrations was measured during 24 h reaction time. Optimal pH, temperature and salt (NaCl) concentration were selected as 7.0, 45 °C and 3.5 M, respectively for the determination of enzyme activity.

*Hmc*Isc enzyme activity was followed as a function of time starting with 150 mM Suc, and reducing sugars were analyzed through the DNS method (Figure 5a). A very long time was required (600 h) before substrate depletion occurred, suggesting a low reaction rate. This was confirmed through a kinetic analysis at increasing Suc concentrations (Figure 5b). One possible explanation for the low reaction rate could be that the enzyme was not expressed in an Archaean expression system (e.g., *Haloferax volcanii*), and this will be considered in the future. However, the enzyme proved stable, and the obtained profiles fitted well with Michaelis-Menten kinetics (GraphPad; R^2^ = 0.9703) with a V_max_ (3.13 µmoles reducing sugar/h/mg protein, i.e., 52 U/g protein or 0.052 U/mg protein) and a K_m_ of 38 ± 3 mM Suc, respectively. Contrastingly, Hill kinetics and much higher V_max_ (296 U/mg protein) K_0.5_ (104.79 mM) values were observed for *Hs*Lsc [25]. Moreover, Michaelis Menten kinetics and very low reaction rates were also observed for the inulosucrase from *Leuconostoc citreum* CW28 (K_m_: 24 mM; V: 0.015 U/mg protein; [73,74]. *L. citreum* accessions are believed to be main players in colonizing the surfaces of the gastrointestinal tract, a long-lasting but highly rewarding process [75]. Very similar kinetics were observed for the *Streptococcus mutans* OMZ175 inulosucrase (K_m_: 17 mM; V: 1.2 U/mg protein; [76]). Both inulosucrases contain atypical N-terminal extensions that may be involved in surface binding processes. *S. mutans* OMZ175 not only contributes to plaque formation and caries in the oral cavity, but its dissemination into the blood stream also increases the risk of heart failure through infection of coronary artery endothelial cells [77]. Taken together, all the above-mentioned organisms are characterized by strong surface binding properties mediated through proteins such as collagen binding proteins [78] and mucin binding proteins [75,79]. It is tempting to speculate that inulins produced by these inulosucrases may also assist in these adherence processes, depending on the access to sufficient Suc as substrate for these enzymes. Assuming such scenario, it can be hypothesized that bacterial niche formation, through surface adherence and time-consuming biofilm formation, including meticulous incorporation of inulin, is a priority rather than rapid Glc-mediated growth. Such a scenario would fit well with the low reaction rates of their Glc-producing inulosucrases.

Assuming that such low reaction rates would also occur for the native *Hmc*Isc, could that be of physiological relevance for *Halomicrobium* spp. in Tuz Lake? These microorganisms are believed to occur in a free-living state in the upper layers of the lake. The only possible access to carbon (Suc) for growth and fructan formation would be through association with a photosynthetic Suc-producing partner [16,23]. Therefore, it may be a matter of uttermost priority for *Halomicrobium* spp. to adhere to such photosynthetic partners (e.g., halophilic *Dunaliella* spp. or cyanobacteria) and establishing such (potentially symbiotic) relationships, which usually depends on a series of signal exchanges between the two partners over time. At extreme salt concentrations (3.5–5.0 M NaCl), biomolecular interactions between the two microorganisms may be much less dependent on specific protein:protein interactions, since this is a rather costly option in terms of ATP requirements. We speculate that Suc-dependent carbohydrate:carbohydrate interactions may become more interesting to serve as the glue between the two organisms. From the perspective of *Halomicrobium*, directly using the energy released from the extracellular splitting of symbiotic Suc to drive fructan synthesis is the energetically cheaper option. Indeed, ATP can be saved by limiting the amount of Suc importers, and especially by avoiding the synthesis of nucleoside diphosphate (NDP)-sugars and lipid carriers that are typically required for the synthesis of other exopolysaccharide types [80]. This may be one reason why fructans are popular within the Haloarchaea but absent in all other Archaea. The natural by-product of fructan biosynthesis, Glc, may be taken up by *Halomicrobium* and used for ATP production, serving growth. Perhaps fructans are relatively more important compared to other exopolysaccharides in Haloarchaea, which remains an interesting topic for future research.

Although extracellular *Hmc*Isc is still enzymatically active at 4.5 M NaCl, the free water at such concentrations becomes extremely scarce. Indeed, although the water binding capacities of fructans and NaCl are very similar [81,82], extreme salt levels will remove the water from the carbohydrates, promoting carbohydrate:carbohydrate interactions. Such processes may occur among *Halomicrobium* spp. themselves or with their photosynthetic partners, potentially forming symbiotic microbial networks. Accordingly, it was observed before that the flocculation rate and exopolysaccharides both increased at increasing salt concentrations in halotolerant bacteria from the plant root context [83]. The steady progression of inulin synthesis may be essential to properly establish such microbial networks. Whether or not this can be linked to the slower enzymatic reaction rates remains an intriguing question for further research. Overall, the variation among reaction rates within GH68 sucrases is enormous [29,65] and references therein], and the reasons for that are poorly understood and never questioned. Likely, enzymatic reaction rates can be linked to the efficiency of the catalytic mechanism, depending on the amino acids in the close vicinity of the catalytic triad and their particular influence on the pKa modulation of the acid-base catalyst [84]. Therefore, it is crucial to obtain deeper insights in the active site functioning of halophilic GH68 sucrases.

### 3.6. Molecular and Structural Analysis

**Figure 6:**
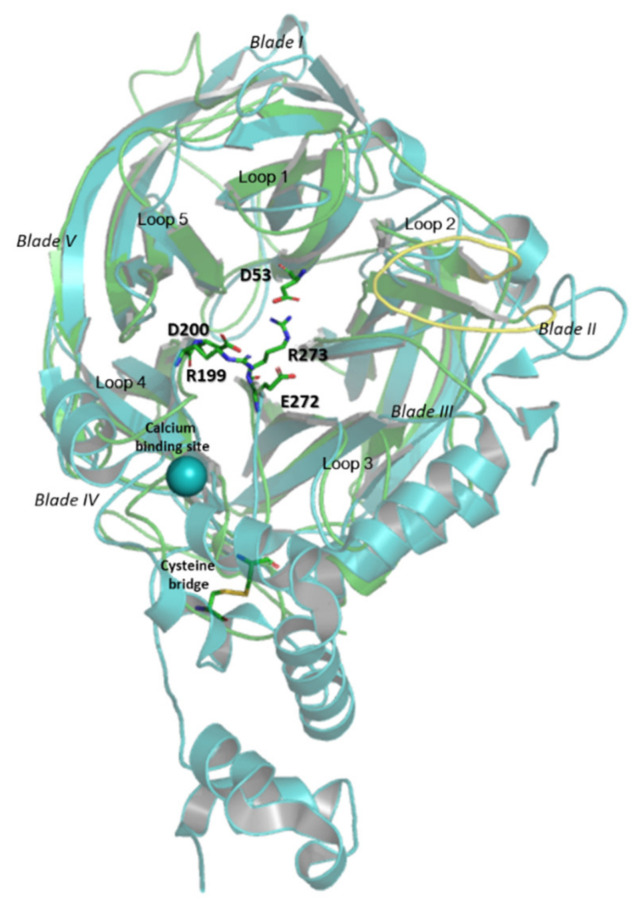
*Hmc*Isc inulosucrase model (green) overlapped with the crystal structure of *L. johnsonii* inulosucrase (2YFR; blue). The root-mean-square deviation (RMSD) between the two structures is 3.839. Loops and blades are indicated, and active site amino acid residues are highlighted. The special Loop 2 in Blade II in *Hmc*Isc is indicated in yellow. *Hmc*Isc shows a disulfide bridge while 2YFR shows a Ca^2+^ binding site.

**Figure 7:**
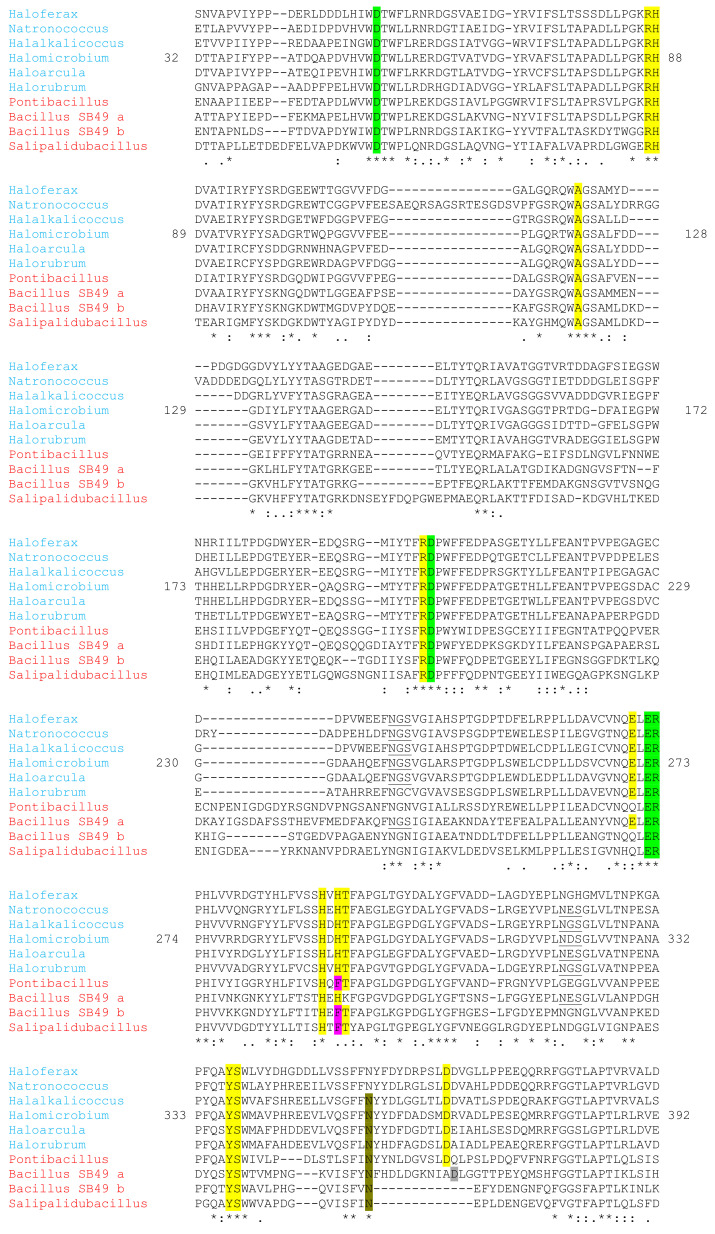
Partial multiple alignment of a number of Haloarchaean (blue) and Gram-positive bacterial (red) GH68 members all showing (potential) inulosucrase signatures. Haloarchaea include *Haloferax gibbonsii* ARA6, *Natronococcus amylolyticus*, *Halalkalicoccus jeotgali*, *Hmc*Isc, *Haloarcula* sp. CBA1115 and *Halorubrum saccharovorum*. Gram-positive bacteria include *Pontibacillus chungwhensis*, *Bacillus* SB49 (variants a and b) and *Salipalidubacillus agaradhaerens.* Predicted canonical N-glycosylation sites are underlined. The three active site acidic residues and the R in the bottom of the active site are in green. Extra amino acids potentially involved in donor and acceptor substrate binding are in yellow, considering F292 (purple) as a functional homologue of H292. The grey D may represent a D365 homologue that did not reach its perfect positioning to assist in acceptor substrate binding. N355 (green-brown) is strictly conserved and may be involved in acceptor substrate binding as well.

A multitemplate model (ITASSER) was produced for *Hmc*Isc with its C-terminal His tag and further minimized according to the optimal working conditions for the enzyme (pH 7.0 and 3.5 M NaCl) and compared to the inulosucrase from *Lactobacillus johnsonii* (2YFS; [34], Figure 6). While the *L. johnsonii* inulosucrase showed a Ca^2+^ binding site, *Hmc*Isc showed a disulfide bridge. Typically, the catalytic triad, consisting of D53 (nucleophile), D200 (transition state stabilizer: R**D**P motif) and E272 (acid-base catalyst: DEI**E**R-like motif) resides within the central deep pocket of the five-bladed propeller (Figure 6). In some of our models, the strictly conserved R273 from the DEIE**R**-like motif (Figure 7) in the bottom of the active site (Figure 6) was found to interact with the nucleophile D53 or the transition state stabilizer D200. However, it is not known whether this observation could be of any physiological importance. As predicted, based on their sequences (see above Section 3.1 and [41]), enzyme surface analysis revealed that the halophilic enzymes *Hmc*Isc and *Hs*Lsc, compared to mesophilic levansucrases *Erwinia amylovora*, *Gluconacetobacter diazotrophicus* (Lsda, [85]), *Bacillus subtilis* (SacB; [86]), and *Lactobacillus johnsonii* inulosucrase [34], are particularly enriched in acidic and hydrophilic residues and also in their active sites (Figure S3). This is a typical characteristic of extracellular halozymes, keeping them fully functional in hypersaline environments. A comparison between the *Hmc*Isc model minimized in water and at 4 M NaCl (RMSD 0.5403) revealed no major changes in its overall structure (not shown). However, no less than 14 salt bridges were observed (Table S3) potentially contributing to the overall enzyme stability.

In the *Hmc*Isc amino acid sequence, two classical NX(S/T) glycosylation sites (N239GS and N320DS) were detected (Figure 7), but the structural analysis revealed that N239 is not accessible for glycosylation. Although nothing is known about glycosylation in *Halomicrobium* sp., a pentasaccharide that is commonly observed in *Haloferax volcanii* (Glucose-(Hexuronic acid)_2_-Methylated hexuronic acid-Mannose; [87]) was inserted on the *Hmc*Isc N320 residue and its mobility was studied (Figure S4). The sugar chain interacted mainly with D31, D32, L234, H395, T396 and I398 but could not reach the active site. Hence, this glycosyl residue is likely involved in enzyme stabilization. Recently, it was uncovered that other glycosylation pathways and glycosylation patterns also exist in *Haloferax volcanii,* some even residing on noncanonical glycosylation sites (Table 2 in [88]). It is, however, not yet clear whether similar, or yet still other, glycosylation patterns occur in other types of Haloarchaea such as *Halomicrobium* spp. Further research on *Hmc*Isc N- and O-glycosylation is warranted, either on the purified native enzyme, if accessible, or on a heterologous enzyme expressed in *H. volcanii* and/or *Pichia pastoris*.

**Figure 8:**
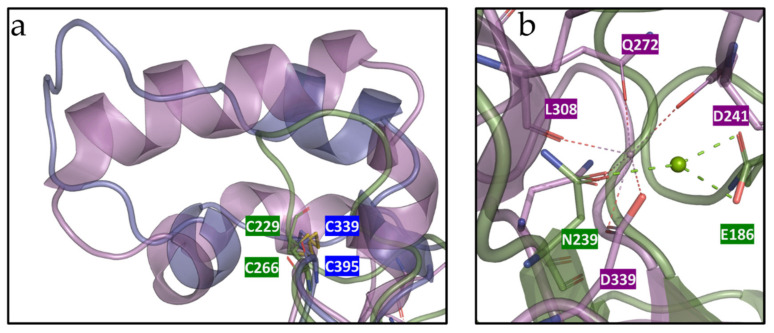
(**a**) Comparison of the *Hmc*Isc inulosucrase model (green) with *B. subtilis* levansucrase 1OYG (purple), and *G. diazotrophicus* levansucrase 1W18 (blue) and the residing cysteine bridges. (**b**) Comparison of the Ca^2+^ binding site in *B. subtilis* levansucrase (purple) and the salt bridge in *Hmc*Isc (green).

Contrary to the 3D structures of *L. johnsonii* inulosucrase [34] and SacB, both containing Ca^2+^ binding sites (D419/Q450/N489/D521 and D241/Q272/N310/D339, respectively) and alpha helix secondary structures (Figure 8a), *Hmc*Isc has none of these. Ca^2+^ binding sites are believed to confer stability to the active site by stabilizing two coil domains that are crucial for the correct functioning of the catalytic triad in a number of GH68 members, with D339 residing in the same loop as the acid-base catalyst E342 (**D**EI**E**R motif) ( [34,86] and references therein). We speculate that the disulfide bound C229-C266 can compensate for the potential loss of stability caused by the absence of a Ca^2+^ binding site in *Hmc*Isc. A similar C339-C395 disulfide bridge was observed at the same location in Lsda [85] which only shows a partial alpha helix secondary structure nearby (Figure 8a). At exactly the same spot as the Ca^2+^ binding site in SacB, a salt bridge between N239 and E186 was observed in *Hmc*Isc, potentially contributing to the well-functioning of the nearby acid-base catalyst E272 (Figure 8b). In conclusion, the combined presence of the disulfide bond and the above-mentioned salt bridge are believed to contribute to *Hmc*Isc stability. Introduction of extra disulfide bridges leads to increased enzyme stability [89] and thermotolerance [90]. This was further corroborated by an in-silico mutagenesis analysis (MOE) on *Hmc*Isc, considering C229A and C266A mutants, leading to a considerable loss in stability (2.85 Kcal/mol for C229A; 5.16 Kcal/mol for C266A).

**Figure 9:**
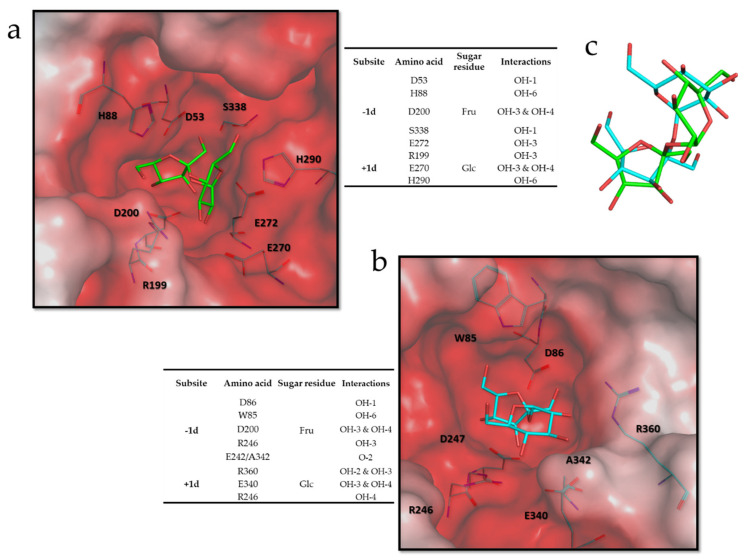
(**a**) Conformation of the Suc binding donor mode obtained by docking analysis with *Hmc*Isc. (**b**) Crystal structure of the Suc donor binding mode in *B. subtilis* levansucrase mutant E342A. (**c**) Overlapping of the Suc conformations in *Hmc*Isc model (blue) and *B. subtilis* levansucrase (green). The incorporated tables show the amino acid interactions as predicted by MOE.

Considering the substrate specificity of *Hmc*Isc, docking analyses (MOE) were performed with Suc as donor substrate and DP6 inulin (GF5) as acceptor substrate. Donor substrate docking revealed that the binding of the Fru part of Suc at the -1 subsite was similar to all previously published poses. Figure 9 compares the situation in *Hmc*Isc (a) and the SacB E342A mutant (b), while an overlay of the two poses is presented in C. The Fru at the -1 donor subsite (-1d) interacts with the nucleophile (D53/D86), the transition state stabilizer (D200/D247), and with a S (S338/S412) residue. When S345, the homologue of S338 in *Hmc*Isc, was mutated to an A residue in *Zymomonas mobilis* levansucrase, an increase in the 1-kestotriose/6-kestotriose product ratio from Suc as only substrate was observed, while the opposite occurred for an A343S mutant in a GH32 member residing in the same operon of that organism [63].

**Figure 10:**
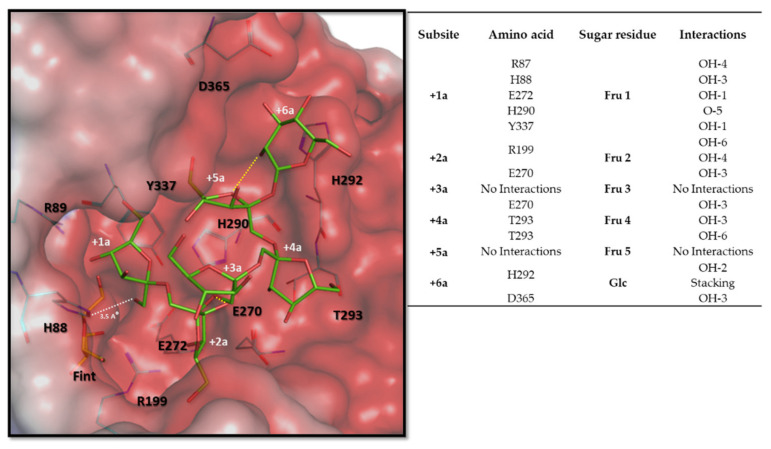
Conformation of the DP6 inulin acceptor binding mode obtained by docking analysis to the *Hmc*Isc inulosucrase fructosyl intermediate. The yellow dashed lines show intramolecular interactions of Fru3 and Fru5, not showing interactions with amino acids. The white dashed line shows the distance of the OH-1 of Fru1 and the anomeric carbon of the fructosyl intermediate (orange). The integrated table shows the amino acid interactions as detected by the molecular operating environment (MOE).

Yet another S to A substitution occurred when comparing *Hmc*Isc and *Hs*Lsc to SacB. S126 from SacB was replaced by A121/A131 in *Hmc*Isc/*Hs*Lsc. Considering a multiple alignment of approved and potential Haloarchean inulosucrases and GH68 members of Gram-positive organisms, their ancestors serving as donors through HGT, the A121 homologue is strictly conserved (Figure 7). Intriguingly, the S126A mutation in SacB already drastically changed the enzyme characteristics, including the synthesis of a blastose oligosaccharide series and a drastic decrease of the turnover number [33]. It would be interesting to investigate whether the opposite mutation A121S in *Hmc*Isc would also result in an opposite effect. Interestingly, the Glc positioning at the +1d subsite takes a different position in *Hmc*Isc (Figure 9a) and SacB E342A (Figure 9b). The binding between Glc and H290 differs from the interaction between Glc and R360 in the SacB E342A. The absence of the acid-base catalyst in the SacB mutant, a strong interactor with Glc in the case of *Hmc*Isc, may also play a role in this differential Glc orientation. After binding Suc as donor substrate, an enzyme-fructosyl intermediate is formed. Estimating as good as possible the exact configuration of this enzyme-fructosyl intermediate is a central and crucial step prior to acceptor substrate docking, predicting the formation of either a β2,1 or a β2,6 linkage. Docking with DP6 inulin, but not with DP6 levan, resulted in a stable, low energy binding configuration that approached the enzyme fructosyl intermediate close enough to form a β2,1 linkage. The terminal Fru5 of the DP6 inulin acceptor shows very poor overlap with the +1d subsite. To avoid confusion, we term this the +1 acceptor (+1a) subsite followed by the +2a till +5a Fru subsites and terminating with the +6a Glc subsite (Figure 10). Intriguingly, this acceptor docking pose (Figure 10) follows a completely different direction than the one revealed for a levan-type oligosaccharide in a SacB D86A/E342A double mutant [91], suggesting that different acceptor binding paths are followed in these two different types of sucrases. Interestingly, our predicted inulin binding path perfectly overlapped with the position of a second Suc molecule in the inulosucrase structure of *L. johnsonii* (2YFS; [34]), but this requires further confirmation through mutagenesis and 3D-structure analysis of *Hmc*Isc and an inulin oligosaccharide as acceptor.

Strikingly, when following the amino acid interactors along the predicted inulin binding path, for most a very prominent conservation was observed among the Haloarchaean (putative) inulosucrases and their closest homologues in Gram-positive bacilli, tentatively termed as the “inulosucrase subfamily” for this purpose (Figure 7). E270 and E272 from the D**E**I**E**R motif, and R199 from the **R**DP motif, all involved in interactions with the Suc donor, also interacted with several Fru residues at different acceptor subsites (Figure 9 and Figure 10). In particular, the +1a Fru also further interacted with R87, H88, H290 and Y337 (Figure 10), conserved within the whole “inulosucrase subfamily” in Haloarchaea (Figure 7). H290 and H88 were also involved in the interaction with the Suc donor substrate and the formation of the *Hmc*Isc fructosyl intermediate (Figure 9 and Figure 10). When N84, the homologue of H88 in *Hmc*Isc, was mutated to a H residue in *Z. mobilis* levansucrase, an increase in the 1-kestotriose/6-kestotriose product ratio from Suc as only substrate was observed, while the opposite occurred for a H79N mutant in a GH32 member residing in the same operon of that organism [63]. This observation indicates that H88 in *Hmc*Isc may be an important player in favouring 1-kestrotriose formation from Suc, and this requires further experimental verification. Most importantly, H292, T293 and D365 are the crucial players to stabilize Fru at the subsites +4a and Glc at the +6a subsites, respectively. Especially the perfect stacking between H292 and the terminal Glc is very striking (Figure 10). Although the Fru residues at subsites +3a and +5a do not interact with the enzyme, O-2 of Fru-3 interacts with OH-3 of Fru2 (distance 2.7 Å) and O-5 of Fru5 interacts with OH-2 of Glc (distance 2.7 Å), contributing to a more stable configuration (Figure 10).

H290, H292, T293 and D365 are also conserved within the Haloarchaea group of the “inulosucrase subfamily” (Figure 7). Thus, we propose that the “HXHT” signature may serve as a good marker for putative Haloarchaean inulosucrases and, by extension, the “HX(H/F)T” marker may be indicative for Gram-positive bacterial inulosucrases. Indeed, modeling of the latter type of enzymes (not shown) revealed that the presence of a F residue can replace H292 to stack with Glc. Focusing on H292 and D365 allows speculation on the possible evolutionary pathways that were followed. In this respect, the extreme halophilic bacterium *Bacillus* sp. SB49 [92] seems very interesting, since one GH68 variant (SB49 a; Figure 7) showed the F292H transition and a long insertion downstream of this locus, not occurring in the SB49 b variant and in the *S. agaradhaerens* inulosucrase (Figure 7). A D365 homologue, although probably already present in the SB49 a insertion, but not yet completely correctly positioned (brown residue; Figure 7), retrieved its optimal position to help stabilize the Glc part of DP6 inulin in the putative *P. chungwhensis* inulosucrase, and from there, after HGT, it reached the Haloarchaea.

## 4. Conclusions

This manuscript describes the first deep characterization of a Haloarchaean inulosucrase adapted to extreme salt environments. We suggest that this enzyme was recruited through HGT from Gram-positive halophilic bacilli and found a widespread family of (potential) inulosucrases carrying typical signatures. Its low substrate conversion rate raised questions on sucrose availability and the physiological function of the generated inulin-type fructans in symbiotic events in salt lakes. By extension, thought provoking questions were raised on the potential roles of inulosucrases and their products in the human body (oral cavity, gut system) and in food fermentation processes. This work suggests that inulosucrases, as compared to levansucrases, are more widespread than originally thought, and have been largely underexplored. Our combined enzymatic and structural analyses provide tools to better discriminate between putative inulosucrases and levansucrases in the future. The discovery of a putative alternative acceptor binding groove will boost further research into product linkage type and degree of polymerization within GH68 and, by extension, in GH32, also comprising plant fructosyltransferases. These fundamental insights will allow, through rational design, the development of novel enzymes that can provide us with novel types of fructans for specific industrial or agronomical applications.

## Figures and Tables

**Table 1:** Effects of two concentrations of various divalent cations and other substances on *Hmc*Isc activity. Error bars represent standard error values for three replicates.

Compound	Activity at 1 mM (%)	Activity at 5 mM (%)
None	100	100
SDS	2.0 ± 0.4	1.0 ± 0.2
Triton X-100	0.5 ± 0.4	1.0 ± 0.5
EDTA	113 ± 6	11.8 ± 12.3
Ba^2+^	108 ± 7	91 ± 17
Fe^2+^	96 ± 9	69 ± 5
Ca^2+^	32 ± 3	0
Cu^2+^	0	0
Co^2+^	0	0
Ni^2+^	0.5 ± 0.7	0
Mg^2+^	52 ± 5	68 ± 9
Mn^2+^	61 ± 9	24 ± 2

## Data Availability

The Whole Genome Shotgun project has been deposited at DDBJ/ENA/GenBank under the accession JADPQC000000000. The version described in this paper is version JADPQC010000000.

## Supplementary Materials

The following are available online at https://www.mdpi.com/article/10.3390/microorganisms9040749/s1, Figure S1: Phylogenetic networks generated in SplitsTree, Figure S2: SDS-PAGE analysis of *Hmc*Isc fractions, Figure S3: Surface analysis of some GH68 models, Figure S4: Glycosylation model of *Hmc*Isc, Table S1: Development of primers for cloning. Table S2: Protein purification table for *Hmc*Isc. Table S3: Sodium ion interactions in the *Hmc*Isc model.
