# Supplementary material for: Functional and Molecular Characterization of the Halomicrobium sp. IBSBa Inulosucrase
Source: Microorganisms. 2021 Apr 2;9(4):749. doi: 10.3390/microorganisms9040749 (PMC8066391; doi:10.3390/microorganisms9040749)
Supplement: Supplementary file 1 [file microorganisms-09-00749-s001.pdf]

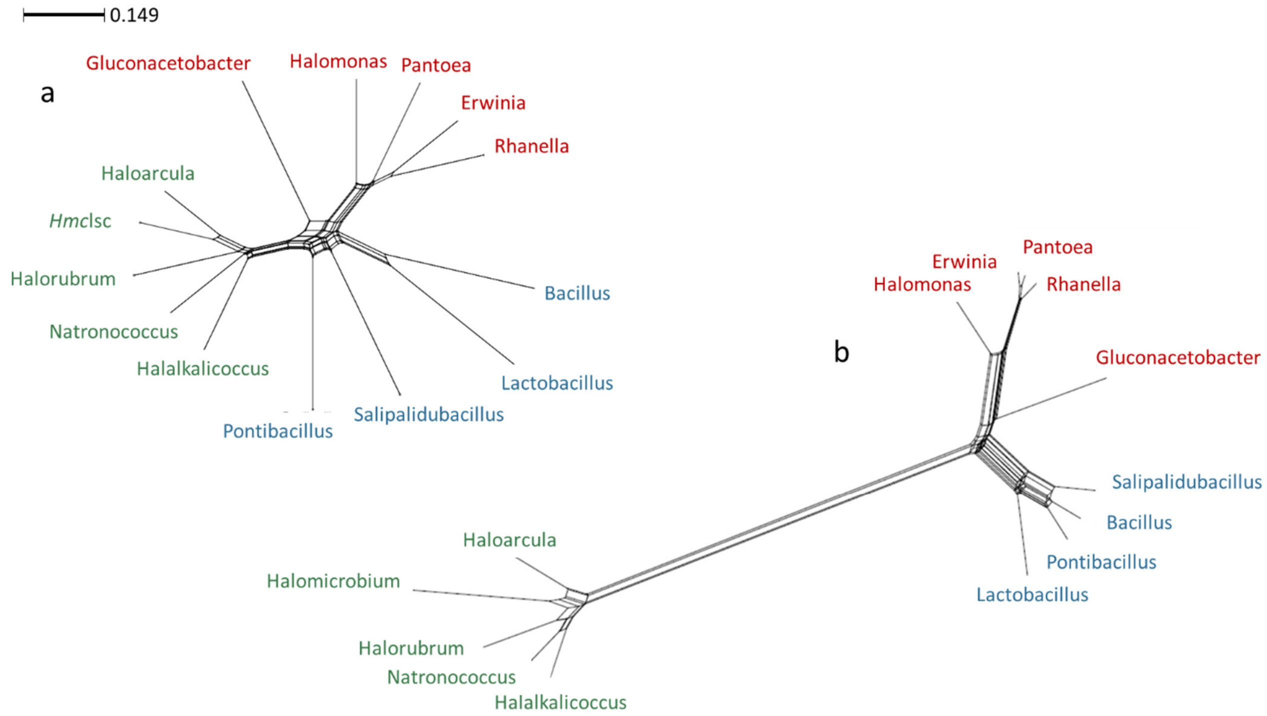


**Figure S1. Phylogenetic networks generated in SplitsTree**. NeighborNet splits analysis based on selected GH68 sequences (a) from Fig. 1a and 16S sequences (b) from Fig. 1b. See Fig. 1 legend for species details. The scale bar indicates 0.00149 substitutions/site.

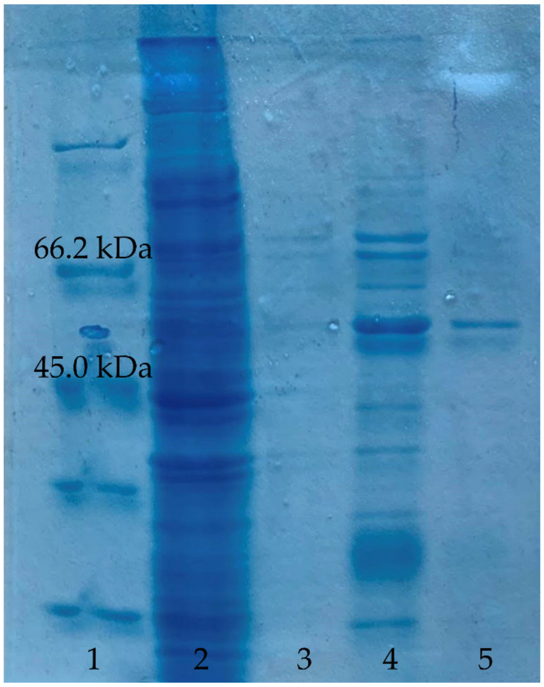


**Figure S2. SDS-PAGE analysis of *Hmc*Isc fractions.** Lanes: **(1)** reference protein ladder, **(2)** induced crude extract, **(3,4,5)** 1st, 2nd (peak fraction) and 3rd fractions of 1 ml each, containing *Hmc*Isc, collected from the IMAC via one-step non-gradient elution with 300 mM imidazole, respectively.

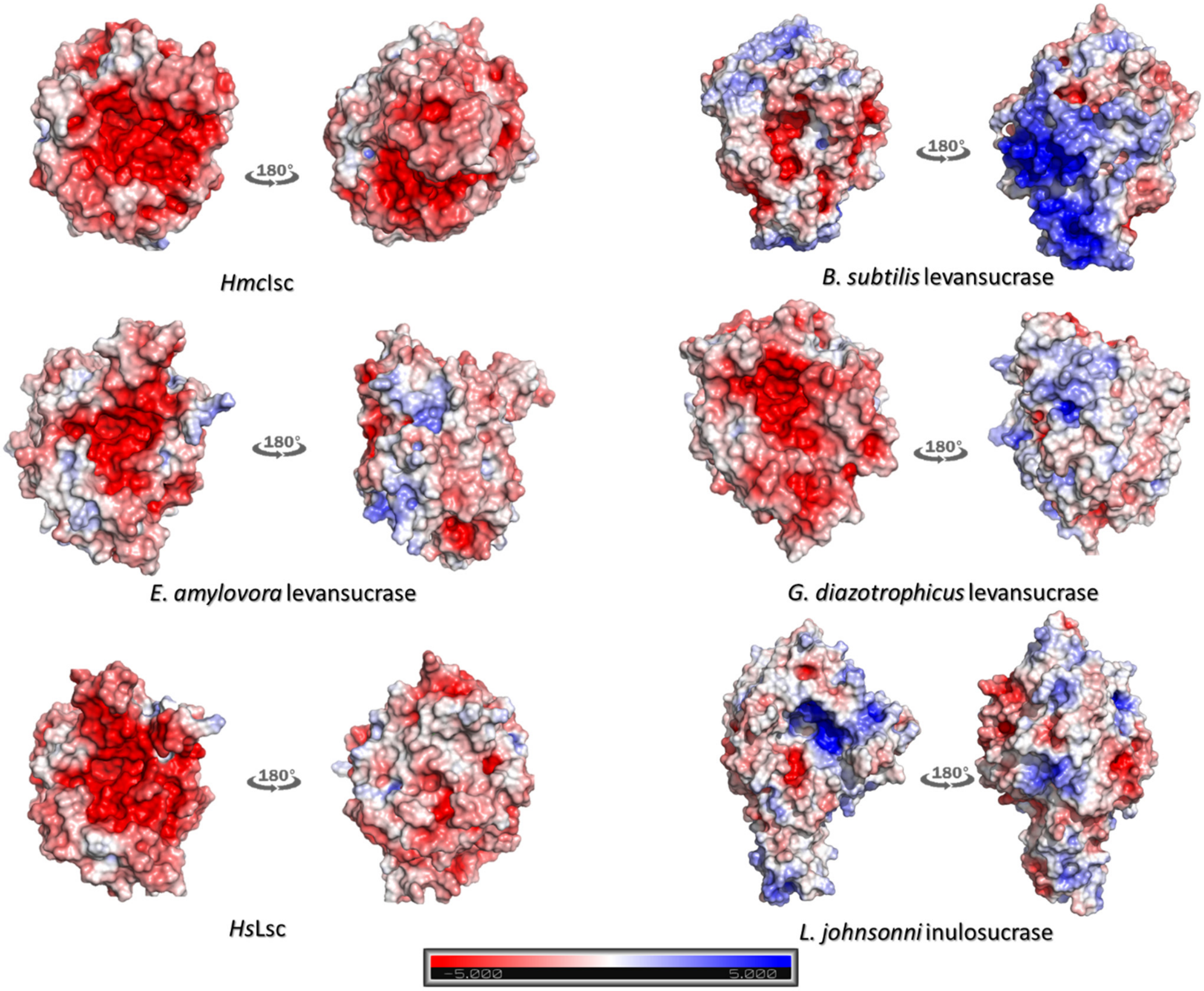


**Figure S3. Surface analysis of GH68 sucrases.** Surface analysis of the *Hmc*Isc inulosucrase model compared to the 3D structures of levansucrases from *B. subtilis* (1OYG), *E. amylovora* (4D47), *G. diazotrophicus* (*1w18*), a model for *H. smyrnensis* levansucrase (*Hs*Lsc) and the inulosucrase from *L. johnsonii* (2YFR). Positively (blue) and negatively charged regions (red) are indicated at pH 7.0.

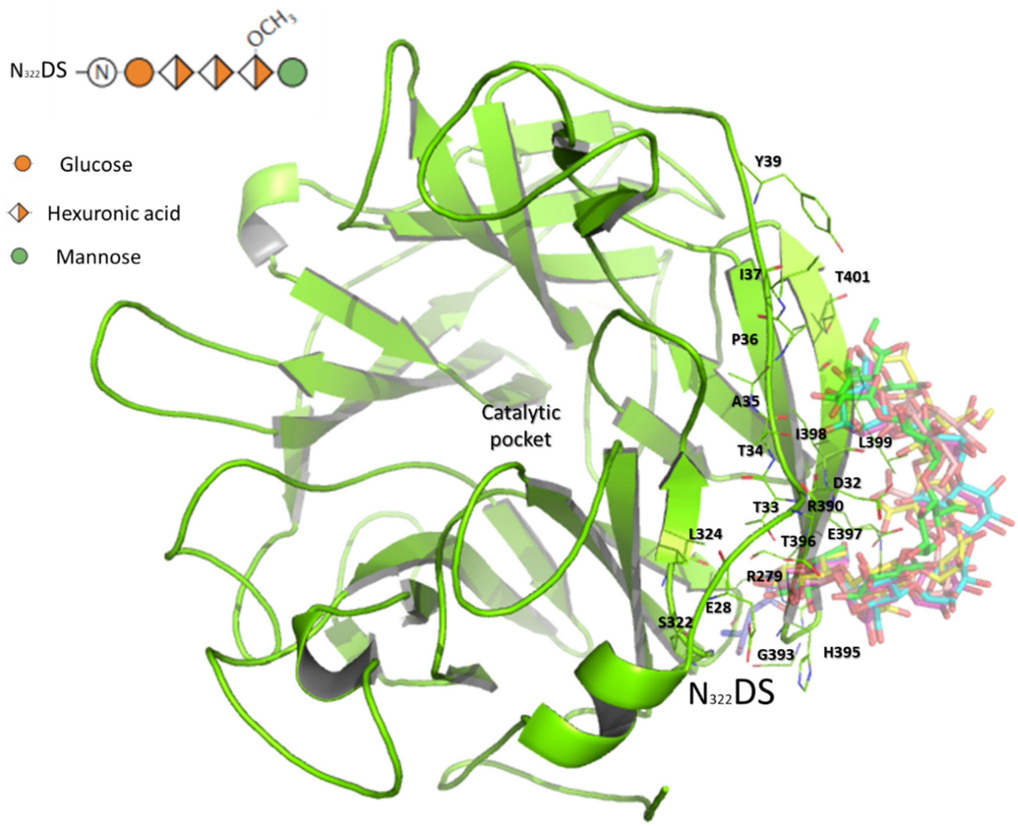


**Figure S4. Glycosylation model of *Hmc*Isc.** Model of the glycosylation on N322 of *Hmc*Isc showing the top 5 conformations of the glycosylation chain as revealed through covalent docking analysis and interaction with neighboring amino acids.

**Table S1. Development of primers for cloning.** Primers designed to clone the *Hmc*Isc gene in frame with the N-terminal and C-terminal His tag. Restriction site consensus sequences are bold and underlined.

| Primer | Sequence (5′ to 3′) |
|---|---|
| C-terminal 6xHis-Tag forward primer | GAT**<u>CCATGG</u>**GCATGAGCAAGGACAGTGCTGG *Nco*I |
| C-terminal 6xHis-Tag reverse primer | GAT**<u>CTCGAG</u>**GCGGCGGTACGATCCG *Xho*I |
| N-terminal 6xHis-Tag forward primer | GAT**<u>CATATG</u>**AGCAAGGACAGTGCTGGG *Nde*I |
| N-terminal 6xHis-Tag reverse primer | GAT**<u>CTCGAG</u>**CTAGCGGCGGTACGATC *Xho*I |
| GH68 forward primer | ATGAGCAAGGACAGTGCTGG |
| GH68 reverse primer | GCGGCGGTACGATCCG |

**Table S2. Protein purification table for *Hmc*Isc.** Comparison of induced and uninduced cell extracts and the purified IMAC peak fraction.

| | Total protein (mg) | Volume (ml) | Total activity (mU) | Specific activity (U/g protein) | Purification (fold) |
|---|---|---|---|---|---|
| Crude cell extract | 50.10 | 5.5 | 56.0±1.3 | 1.1** | 1 |
| IMAC (peak fraction*) | 0.54 | 1.0 | 30.5±1.0 | 56.5±2.0 | 51.4 |
| Uninduced cell extract (control) | 23.59 | 5.5 | 2.3±0.3 | 0.1** | - |

*Peak fraction refers to the protein fraction on lane 4 on SDS-PAGE (Fig. S2).
**Standard error values for specific activities of crude cell extracts are below 0.02.

**Table S3. Sodium ion interactions in the *Hmc*Isc model**. Interactions of sodium ions at 4 M concentration with amino acid side chains and backbones of *Hmc*Isc protonated at pH 7.0. Each interaction represents one different $Na^+$ atom interacting with the respective amino acids. The interactions were measured and calculated using MOE. The total energy of all the interactions is -399.62 Kcal/mol.

| Interactions with amino acid side chains | | | |
|---|---|---|---|
| **Interaction** | **Amino acid** | **Energy Kcal/mol** | **Distance A°** |
| *1* | D53 | -31.88 | 2.34 |
| | E272 | -13.15 | 2.33 |
| | **Total** | **-45.03** | |
| *2* | D302 | -18.11 | 2.22 |
| | D291 | -15.51 | 2.35 |
| | P333 | -3.48 | 2.34 |
| | **Total** | **-37.1** | |
| *3* | E186 | -28.18 | 2.37 |
| | N239 | -1.53 | 2.45 |
| | **Total** | **-29.71** | |
| *4* | D127 | -27.11 | 2.32 |
| | E59 | -19.09 | 2.22 |
| | F126 | -2.35 | 2.3 |
| | **Total** | **-48.55** | |
| *5* | E425 | -26.19 | 2.36 |
| | T159 | -2.3 | 2.45 |
| | **Total** | **-28.49** | |
| *6* | D365 | -16.66 | 2.32 |
| | H292 | -4.26 | 2.45 |
| | **Total** | **-20.92** | |
| *7* | D48 | -20.2 | 2.62 |
| | P47 | -1.84 | 2.43 |
| | D44 | -1.49 | 3.61 |
| | **Total** | **-23.53** | |
| *8* | D4 | -25.39 | 2.41 |
| | Q23 | -3.3 | 2.47 |
| | G18 | -2.45 | 2.36 |
| | **Total** | **-31.14** | |
| **Interactions with amino acid backbones** | | | |
| **Interaction** | **Amino acid** | **Energy Kcal/mol** | **Distance A°** |
| *9* | D291 | -3.42 | 2.42 |
| | L298 | -3.15 | 2.35 |

| | | | |
|---|---|---|---|
| | F294 | -1.55 | 2.37 |
| | **Total** | **-8.12** | |
| *10* | R434 | -29.95 | 2.39 |
| | V109 | -3.02 | 2.43 |
| | V110 | -0.87 | 2.7 |
| | **Total** | **-33.84** | |
| *11* | D360 | -19.41 | 2.23 |
| | E374 | -3.49 | 2.31 |
| | A361 | -3.29 | 2.38 |
| | **Total** | **-26.19** | |
| *12* | E113 | -18.73 | 2.21 |
| | F111 | -2.78 | 2.31 |
| | E112 | -1.24 | 2.35 |
| | **Total** | **-22.75** | |
| *13* | E211 | -3.98 | 2.38 |
| | F204 | -3.15 | 2.34 |
| | H213 | -2.52 | 2.38 |
| | **Total** | **-9.65** | |
| *14* | D413 | -32.05 | 2.31 |
| | L414 | -2.55 | 2.37 |
| | **Total** | **-34.6** | |
